# Associations of Inflammatory Prognosis Index With All‐Cause and Cardiovascular Mortality Among Individuals With Diabetes and Prediabetes: A Retrospective Cohort Study

**DOI:** 10.1155/mi/6052273

**Published:** 2026-03-28

**Authors:** Lu Liu, Jiasuer Alifu, Abdul-Quddus Mohammed, Guoqing Yin, Fuad A. Abdu, Wenliang Che

**Affiliations:** ^1^ Department of Cardiology, Shanghai Tenth People’s Hospital, Tongji University School of Medicine, Shanghai, China, tongji.edu.cn; ^2^ Department of Cardiology, Shanghai Tenth People’s Hospital, Chongming Branch, Shanghai, China, shdsyy.com.cn

**Keywords:** diabetes, inflammatory biomarkers, mortality, NHANES, prediabetes

## Abstract

**Background:**

The systemic immune‐inflammatory index (SII) and the systemic inflammatory response index (SIRI) have shown prognostic value in diabetes mellitus (DM). However, the impact of the inflammatory prognosis index (IPI) remains unexamined. This retrospective cohort study investigates the predictive value of IPI, along with SIRI and SII, on all‐cause and cardiovascular disease (CVD) mortality in individuals with DM and prediabetes mellitus (pre‐DM).

**Method:**

We analyzed DM and pre‐DM individuals from the National Health and Nutrition Examination Survey (NHANES) (1999–2018), with follow‐up conducted until December 31, 2019. Cox models assessed the correlation of IPI, SIRI, and SII with mortalities, yielding hazard ratios (HRs) and 95% confidence intervals (CIs). We also utilized restricted cubic spline (RCS) analysis, Kaplan–Meier curves, and subgroup analyses.

**Results:**

The final analysis included 10,049 individuals with DM and pre‐DM. Over a mean follow‐up of 9 years, 2233 all‐cause mortalities occurred, with 612 attributed to CVD. Fully adjusted Cox regression analysis revealed a significant correlation between continuous and higher tertiles (T3) of IPI, SIRI, and SII and increased risk of all‐cause and CVD mortality across all models (all *p*  < 0.05). Kaplan–Meier curves showed higher IPI, SIRI, and SII associated with elevated mortality risk (all log‐rank *p*  < 0.001). RCS analysis demonstrated a nonlinear correlation between IPI and all‐cause and CVD mortality, as well as between SII and all‐cause mortality (nonlinear *p*  < 0.001). However, SIRI exhibited a linear association with both all‐cause and CVD mortality.

**Conclusion:**

Elevated IPI, along with SIRI and SII, is linked to increased all‐cause and CVD‐related mortality in DM and pre‐DM, suggesting that incorporating IPI in routine screening could help identify high‐risk individuals for earlier interventions.

## 1. Introduction

Diabetes mellitus (DM) presents a significant global health concern, impacting over 460 million people and imposing substantial burdens on healthcare systems worldwide, with annual increases projected [1]. Prediabetes mellitus (pre‐DM), defined by impaired glucose tolerance, fasting glucose, or hemoglobin A1c (HbA1c) levels, affects a larger population than DM itself, with 34.5% of US adults [2] and 35.7% of Chinese adults reported to have pre‐DM [3]. Both conditions are established risk factors for cardiovascular disease (CVD) and are associated with elevated all‐cause and CVD mortality [4–8]. Preventive strategies, including early intervention and identification of high‐risk individuals using inflammatory biomarkers, can delay disease progression, reduce cardiovascular and inflammatory risks, and guide anti‐inflammatory pharmacological treatments to improve long‐term outcomes.

Inflammations are crucial players in the complex relationship between DM development and progression, serving as a critical mediator in the pathogenesis of this chronic metabolic disorder [9, 10]. Novel systemic inflammatory markers, such as the systemic inflammatory response index (SIRI) and systemic immune‐inflammation index (SII), are predictive indices that may provide a reliable and practical method for prognosis [11–13]. These biomarkers have emerged as reliable predictors of DM development, cardiovascular complications, and long‐term mortality in diabetic populations [14–21]. The inflammatory prognosis index (IPI), calculated as C‐reactive protein (CRP) × neutrophil‐to‐lymphocyte ratio (NLR)/albumin, has been studied as a prognostic marker in various clinical settings [11, 22, 23]. However, its prognostic role in individuals with DM and pre‐DM remains unexplored, representing a critical gap in the current evidence.

Therefore, this study aimed to investigate the association between IPI levels and long‐term all‐cause and CVD mortality in individuals with DM and pre‐DM using data from the National Health and Nutrition Examination Survey (NHANES). Evaluating this previously unreported biomarker may provide a valuable tool for risk stratification and enable earlier interventions to better manage at‐risk patients.

## 2. Methods

### 2.1. Study Design and Population

This study is a retrospective cohort analysis using prospectively collected, nationally representative data from NHANES. NHANES is an ongoing cross‐sectional survey conducted by the National Center for Health Statistics (NCHS) to assess the health and nutritional status of the noninstitutionalized civilian US population. We formed a closed cohort by linking participants’ baseline data, including inflammatory indices, diabetes status, and other covariates, from NHANES examination cycles (1999–2018) with prospective mortality follow‐up data through the National Death Index (NDI) until December 31, 2019. This method, which uses a baseline survey to define a cohort followed prospectively for definitive endpoints, is classified as a retrospective cohort study when analyzed later. The analytic cohort consisted of adults aged 18 years or older who had been diagnosed with DM or pre‐DM at their baseline NHANES examination.

**Figure 1:**
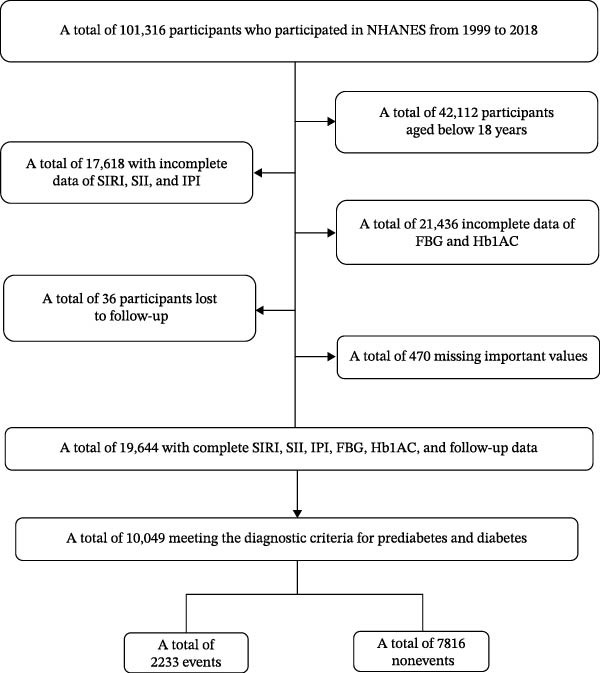
National Health and Nutrition Examination Survey (NHANES) recruitment flow diagram 1999–2018.

This investigation collected data from 101,316 participants from 1999 to 2018. Exclusions were made for individuals under the age of 18 (*n* = 42,112), incomplete data on SIRI, SII, and IPI (including monocyte counts, platelet counts, albumin, neutrophil counts, lymphocyte counts, and CRP (*n* = 17,618), incomplete data on fasting blood glucose (FBG) and HbA1c (*n* = 21,436), loss to follow‐up (*n* = 36), missing important values such as hypertension, chronic kidney disease (CKD), heart failure (HF), stroke, and coronary heart disease (CHD) (*n* = 470), and those not meeting the diagnostic criteria for pre‐DM and DM (*n* = 9595). Following the selection process, 10,049 individuals diagnosed with DM and pre‐DM were included, with 2233 experiencing all‐cause mortalities (Figure 1).

All surveys underwent thorough review and approval by the NCHS Ethics Review Committee, and all participants provided written informed consent prior to participation.

### 2.2. Definition of DM and Pre‐DM

Pre‐DM was determined through self‐reported pre‐DM status, FBG levels between 5.6 and 6.9 mmol/L, or HbA1c levels between 5.7% and 6.4% [24]. Any of the following criteria define DM: a self‐reported diagnosis, FBG levels ≥ 7 mmol/L, HbA1c levels ≥ 6.5%, random blood glucose levels ≥ 11.1 mmol/L, a 2 h oral glucose tolerance test blood glucose level ≥ 11.1 mmol/L, or the use of antidiabetic medications or insulin [24].

### 2.3. Calculation of Inflammatory Biomarkers

We obtained laboratory measurements for platelets, albumin, neutrophils, monocytes, lymphocytes, and CRP data. Complete blood cell counts, including platelets, neutrophils, monocytes, and lymphocytes, were performed on EDTA‐anticoagulated whole blood. Albumin and CRP were measured in serum samples. These values were employed in computing the following inflammatory biomarkers: (1) SII is calculated as platelet count × NLR; (2) SIRI is determined by neutrophils × monocytes count divided by lymphocyte count; and (3) IPI is determined as CRP × NLR/albumin [11]. All components for these indices were derived from a single baseline blood sample, ensuring internal consistency. It is important to note that the NHANES protocol did not specifically screen for or exclude participants based on acute inflammatory conditions (e.g., active infection) or chronic localized inflammation (e.g., diabetic foot ulcers) at the time of measurement.

### 2.4. Assessment of Covariates

Participants’ demographic information, including gender, age, ethnicity, and educational attainment, was obtained from the NHANES demographic questionnaire. Race/ethnicity classifications comprised Mexican American, non‐Hispanic White/Black, and other Hispanic Asian/multiracial groups. Education status was simplified into three categories: lower than high school, high school diploma, and greater than high school. Smoking status is defined as never or current smoking. Information regarding a history of hypertension, CKD, HF, stroke, and CHD was collected through self‐report questionnaires. Additionally, various laboratory counts such as white blood cell (WBC), neutrophil, lymphocyte, monocyte, and platelet, as well as levels of albumin, CRP, FBG, HbA1c, triglyceride (TG), total cholesterol (TC), high‐density lipoprotein cholesterol (HDL‐C), low‐density lipoprotein cholesterol (LDL‐C), aspartate aminotransferase (AST), serum creatinine, and alanine aminotransferase (ALT), were assessed in the NHANES laboratory.

### 2.5. Assessment of Mortality

Our prognosis analysis considered all‐cause and CVD‐related mortality as primary endpoints. All‐cause mortality is identified using publicly available death data from NHANES and the NDI before December 31, 2019. All‐cause mortality was defined according to the International Classification of Diseases, Tenth Revision (ICD‐10), encompassing deaths from heart disease, chronic lower respiratory diseases, DM, and all other causes. Death attributed to CVD was delineated by ICD‐10 codes I00‐I09, I11, I13, I20‐I51, or I60‐I69.

Data on mortality variables can be accessed via the following website: https://www.cdc.gov/nchs/data-linkage/mortality.htm.

## 3. Statistical Analyses

Statistical analyses were conducted using various software packages to ensure robustness and appropriate methodology. Primary analyses, including data management, descriptive statistics, and the main Cox proportional hazards regression, were performed with the Statistical Package for the Social Sciences (SPSS) v.25. Figures were created using GraphPad Prism Version 9. For advanced modeling, such as restricted cubic spline (RCS) analysis and other specialized procedures, we used R software (version 4.2.2, R Foundation for Statistical Computing, Vienna, Austria), along with MSTATA software (https://www.mstata.com/). Continuous variables are reported as mean ± standard deviation (SD) for normally distributed data or as median with interquartile range (IQR) for nonnormally distributed data. Categorical variables are shown as frequencies (%). Intergroup comparisons were conducted using the independent samples *t*‐test (or Welch’s *t*‐test when variances were unequal) for normally distributed continuous variables, the Wilcoxon rank‐sum test for nonnormally distributed continuous variables, and the chi‐square test (or Fisher’s exact test where appropriate) for categorical variables. Cox proportional models investigated the relationships between SIRI, SII, and IPI and all‐cause mortality and CVD‐related mortality, estimating hazard ratios (HRs) and 95% confidence intervals (95% CIs). Three models were constructed: a crude model without adjustment; Model 1 adjusted for age, sex, race, education status, BMI, and smoking status; and Model 2 further adjusted for CHD, CKD, HF, stroke, hypertension, TC, TG, HDL, LDL, ALT, AST, and serum creatinine. To explore potential nonlinear relationships, RCS analyses were performed for each inflammatory index in relation to both mortality outcomes. The RCS models were adjusted for the same comprehensive set of covariates as in Model 2. For each index‐outcome pair, models with 3–7 knots were tested. The optimal number and placement of knots were selected based on the lowest Akaike information criterion (AIC). Final knot locations (percentiles) were as follows: SIRI with all‐cause mortality (five knots: 5th, 28th, 50th, 72nd, and 95th) and CVD mortality (four knots: 5th, 35th, 65th, and 95th); SII with all‐cause mortality (seven knots: 2nd, 18th, 34th, 50th, 66th, 82nd, and 98th) and CVD mortality (three knots: 10th, 50th, and 90th); and IPI with both outcomes (three knots: 10th, 50th, and 90th). The overall association (*p*‐overall) and nonlinearity (*p*‐nonlinear) were assessed using these models. The Kaplan–Meier analysis compared survival‐free from all‐cause and CVD death, with differences assessed using log‐rank tests. A Cox proportional hazards model was used to examine the association between SIRI, SII, and IPI across patient subgroups defined by age (<65 years vs. ≥65 years), sex (male vs. female), hypertension, CHD, and CKD, with an interaction term included to assess heterogeneity between subgroups. To verify the stability of our results, sensitivity analysis was performed by excluding participants who deceased within 2 years of follow‐up. All analyses were performed as two‐sided tests, and statistical significance is set when the *p*‐value is <0.05.

## 4. Results

Following the selection process, 10,049 individuals meeting the diagnostic criteria for DM and pre‐DM were included in the final analysis of the present study (Figure 1), with a mean age of 56.33 ± 16.49 years and males comprising 54.2% of the sample.

### 4.1. Baseline Characteristics of Participants According to Survival Status

Table 1 presents the clinical characteristics and laboratory parameters of the study population, categorized by all‐cause mortality status. Individuals who experienced mortalities were older, more likely to be male, had a higher proportion of Mexican American ethnicity, and had a higher prevalence of smoking. They also exhibited higher systolic blood pressure and were more prone to comorbidities such as hypertension, HF, stroke, CKD, and CHD compared to nonevent individuals. Laboratory parameters, including WBC, neutrophil, and monocyte counts, were higher in the events group, whereas platelet counts, CRP, and lymphocyte counts were lower. Importantly, systemic inflammatory biomarkers, including SIRI, SII, and NLR, were notably higher in the events group (*p*  < 0.05). Significant differences were also observed in other parameters, including FBG, HbA1c, LDL cholesterol, serum creatinine, and ALT, between the two groups (all *p*  < 0.05).

**Table 1:** Clinical characteristics of the study population stratified by all‐cause mortality status.

	Events (*N* = 2233)	Nonevents (*N* = 7816)	*p*‐Value
General characteristics			
Age (years)	69.99 ± 11.901	52.43 ± 15.51	<0.001
BMI (kg/m^2^)	29.24 ± 6.43	30.63 ± 7.11	<0.001
Male, *n* (%)	1290 (57.8)	4161 (53.2)	<0.001
SBP (mmHg)	136.76 ± 23.07	127.11 ± 18.48	<0.001
DBP (mmHg)	65.79 ± 16.93	71.45 ± 12.94	<0.001
Race/ethnicity, *n* (%)			<0.001
Mexican American	673 (30.1)	1872 (24.0)	—
Non‐Hispanic White	206 (9.2)	969 (12.4)	—
Non‐Hispanic Black	1002 (44.9)	2786 (35.6)	—
Other Hispanic/Asian/multiracial	352 (15.8)	2189 (28.0)	—
Education levels, *n* (%)			<0.001
Less than high school	914 (40.9)	2308 (29.5)	—
High school diploma	561 (25.1)	1835 (23.5)	—
More than high school	758 (34.0)	3673 (47.0)	—
Smoking status, *n* (%)			<0.001
No	880 (39.4)	4157 (53.2)	—
Yes	1353 (60.6)	3659 (46.8)	—
Comorbidities			
Hypertension, *n* (%)	1355 (60.7)	3278 (41.9)	<0.001
Heart failure, *n* (%)	269 (12.0)	205 (2.6)	<0.001
Stroke, *n* (%)	256 (11.5)	269 (3.4)	<0.001
CKD, *n* (%)	160 (7.2)	258 (3.3)	<0.001
CHD, *n* (%)	276 (12.4)	332 (4.2)	<0.001
Laboratory parameters			
WBC counts (10^9^/L)	6.90 (5.70, 8.40)	6.60 (5.60, 8.00)	<0.001
Neutrophil counts (10^9^/L)	4.20 (3.30, 5.30)	3.80 (3.00, 4.80)	<0.001
Lymphocyte counts (10^9^/L)	1.80 (1.40, 2.30)	2.00 (1.60, 2.40)	<0.001
Monocyte counts (10^9^/L)	0.60 (0.50, 0.70)	0.50 (0.40, 0.60)	<0.001
Platelet counts (10^9^/L)	237.00 (196.00, 287.00)	244.00 (206.00, 290.00)	<0.001
Albumin (g/L)	41.00 (39.00, 44.00)	42.00 (40.00, 44.00)	<0.001
CRP (mg/dL)	0.36 (0.15, 0.82)	0.54 (0.18, 1.80)	<0.001
SIRI	1.30 (0.89, 1.96)	0.98 (0.66, 1.44)	<0.001
SII	545 (377, 807)	466 (330, 653)	<0.001
IPI	0.02 (0.01, 0.05)	0.02 (0.01, 0.09)	<0.001
NLR	2.32 (1.69, 3.20)	1.90 (1.43, 2.56)	<0.001
FBG (mmol/L)	7.23 ± 2.94	6.67 ± 2.26	<0.001
Hb1AC (%)	6.33 ± 1.40	6.06 ± 1.24	<0.001
TC (mmol/L)	5.09 ± 1.16	5.10 ± 1.09	0.470
HDL‐C (mmol/L)	1.24 (1.03, 1.55)	1.27 (1.06, 1.53)	0.534
LDL‐C (mmol/L)	2.85 (2.25, 3.57)	3.03 (2.41, 3.65)	<0.001
TG (mmol/L)	1.45 (0.99, 2.08)	1.34 (0.94, 1.96)	<0.001
Serum creatinine (umol/L)	82.21 (70.70, 106.08)	73.37 (61.88, 88.40)	<0.001
AST (U/L)	23 (19, 28)	23 (19, 28)	0.151
ALT (U/L)	20 (15, 26)	22 (17, 31)	<0.001

Abbreviations: ALT, alanine aminotransferase; AST, aspartate aminotransferase; BMI, body mass index; CHD, coronary heart disease; CKD, chronic kidney disease; CRP, C‐reactive protein; DBP, diastolic blood pressure; FBG, fasting blood glucose; HbA1c, hemoglobin A1c; HDL‐C, high‐density lipoprotein‐cholesterol; IPI, inflammatory prognostic index; LDL‐C, low‐density lipoprotein‐cholesterol; NLR, neutrophil‐to‐lymphocyte ratio; SBP, systolic blood pressure; SII, systemic immune‐inflammation index; SIRI, systemic inflammation response index; TC, total cholesterol; TG triglyceride; WBC, white blood cell.

### 4.2. Associations of IPI, SIRI, and SII With All‐Cause and CVD Mortality

Tables 2 and 3 display HR with 95% CI for all‐cause and CVD‐related mortality across three models, categorized by SIRI, SII, and IPI tertiles. In the SIRI analysis, both continuous SIRI values and categorical tertiles demonstrated a significant association with all‐cause and CVD mortality risk in all models, with higher values indicating increased risk. T3 exhibited progressively higher mortality risk than the other tertiles, with significant trends across tertiles. Similarly, in the SII analysis, both continuous SII values and categorical tertiles were significantly associated with all‐cause and CVD mortality risk in all models, particularly in tertile 3. However, the association for tertile 2 was not consistently significant across all adjusted models. The IPI analysis revealed that both continuous categorical tertiles were significantly associated with all‐cause and CVD mortality risk in all models, with tertiles 2 and 3 showing higher risks than tertile 1. Overall, despite some variability in the significance of individual tertiles, a statistically significant increasing trend in mortality risk across tertiles was observed for all three biomarkers (all *p* for trend <0.05).

**Table 2:** Hazard ratios (95% confidence intervals) for all‐cause mortality according to the SIRI, SII, and IPI.

	Crude model		Model 1		Model 2	
	HR (95% CI)	*p*‐Value	HR (95% CI)	*p*‐Value	HR (95% CI)	*p*‐Value
SIRI						
SIRI (continuous)	1.301 (1.273–1.328)	<0.001	1.227 (1.189–1.265)	<0.001	1.189 (1.149–1.230)	<0.001
T1	—	reference	—	reference	—	reference
T2	1.515 (1.346–1.704)	<0.001	1.201 (1.063–1.358)	0.003	1.161 (1.021–1.321)	0.023
T3	2.616 (2.346–2.917)	<0.001	1.643 (1.459–1.849)	<0.001	1.457 (1.287–1.651)	<0.001
*p* for trend	—	<0.001	—	<0.001	—	<0.001
SII						
SII (continuous)	1.000(1.000–1.000)	<0.001	1.000 (1.000–1.000)	<0.001	1.000 (1.000–1.000)	<0.001
T1	—	reference	—	reference	—	reference
T2	1.013(0.907–1.132)	0.815	0.930 (0.829–1.043)	0.213	0.954 (0.846–1.076)	0.446
T3	1.460(1.318–1.618)	<0.001	1.243 (1.116–1.385)	<0.001	1.213 (1.084–1.359)	0.001
*p* for trend	—	<0.001	—	<0.001	—	<0.001
IPI						
IPI (continuous)	1.072 (1.049–1.096)	<0.001	1.086 (1.059–1.114)	<0.001	1.084 (1.054–1.115)	<0.001
T1	—	reference	—	reference	—	reference
T2	1.381(1.254–1.521)	<0.001	1.241 (1.122–1.373)	<0.001	1.250 (1.124–1.389)	<0.001
T3	1.811(1.623–2.021)	<0.001	1.881 (1.672–2.117)	<0.001	1.752 (1.545–1.986)	<0.001
*p* for trend	—	<0.001	—	<0.001	—	<0.001

*Note:* Crude model: There are no covariates were adjusted. Model 1: Age, sex, race, education level, BMI, and smoking status were adjusted. Model 2: Model 1 + CHD, CKD, HF, stroke, hypertension, TC, TG, HDL, LDL, ALT, AST, and serum creatinine were adjusted.

Abbreviations: CI, confidence interval; HR, hazard ratio; IPI, inflammatory prognostic index; SII, systemic immune‐inflammation index; SIRI, systemic inflammation response index.

**Table 3:** Hazard ratios (95% confidence intervals) for CVD mortality according to the SIRI, SII, and IPI.

	Crude model		Model 1		Model 2	
	HR (95% CI)	*p*‐Value	HR (95% CI)	*p*‐Value	HR (95% CI)	*p*‐Value
SIRI						
SIRI (continuous)	1.317 (1.269–1.367)	<0.001	1.262 (1.195–1.332)	<0.001	1.217 (1.146–1.293)	<0.001
T1	—	Reference	—	Reference	—	Reference
T2	1.659 (1.311–2.099)	<0.001	1.298 (1.015–1.660)	0.037	1.226 (0.946–1.589)	0.124
T3	3.149 (2.539–3.907)	<0.001	1.982 (1.568–2.504)	<0.001	1.696 (1.323–2.173)	<0.001
*p* for trend	—	<0.001	—	<0.001	—	<0.001
SII						
SII (continuous)	1.000 (1.000–1.000)	<0.001	1.000 (1.000–1.000)	<0.001	1.000 (1.000–1.000)	0.001
T1	—	Reference	—	Reference	—	Reference
T2	1.231 (0.994–1.524)	0.056	1.153 (0.923–1.441)	0.210	1.240 (0.980–1.570)	0.073
T3	1.604 (1.309–1.964)	<0.001	1.404 (1.133–1.738)	0.002	1.374 (1.094–1.726)	0.006
*p* for trend	—	<0.001	—	0.001	—	0.007
IPI						
IPI (continuous)	1.071 (1.026–1.118)	0.002	1.085 (1.038–1.135)	<0.001	1.209 (1.113–1.312)	0.002
T1	—	Reference	—	Reference	—	Reference
T2	1.525 (1.264–1.839)	<0.001	1.323 (1.087–1.610)	0.005	1.271 (1.033–1.564)	0.023
T3	2.055 (1.667–2.535)	<0.001	2.115 (1.688–2.650)	<0.001	1.981 (1.560–2.515)	<0.001
*p* for trend	—	<0.001	—	<0.001	—	<0.001

*Note:* Crude model: There are no covariates were adjusted. Model 1: Age, sex, race, education level, BMI, and smoking status were adjusted. Model 2: Model 1 + CHD, CKD, HF, stroke, hypertension, TC, TG, HDL, LDL, ALT, AST, and serum creatinine were adjusted.

Abbreviations: CI, confidence interval; HR, hazard ratio; IPI, inflammatory prognostic index; SII, systemic immune‐inflammation index; SIRI, systemic inflammation response index.

The Kaplan–Meier curve (Figure 2) supports the above findings by indicating that individuals in the higher SIRI (T3 group) face an increased risk of primary endpoints (both all‐cause and CVD mortality) over a median follow‐up of 117 (42–158) months compared with lower SIRI levels (all log‐rank *p*  < 0.001) (Figure 2A,B). Similarly, individuals in the higher SII (T3 group) exhibit an elevated risk of primary endpoints compared to those with lower SII levels (all log‐rank *p*  < 0.001) (Figure 2C,D). Additionally, individuals in the higher IPI (T3 group) also demonstrated an elevated risk of both all‐cause and CVD mortality compared with lower IPI levels (all log‐rank *p*  < 0.001) (Figure 2E,F).

Figure 2Survival free from all‐cause mortality categorized by SIRI, SII, and IPI (A, C, and E), and survival free from CVD mortality categorized by SIRI, SII, and IPI (B, D, and F).

**Figure:**
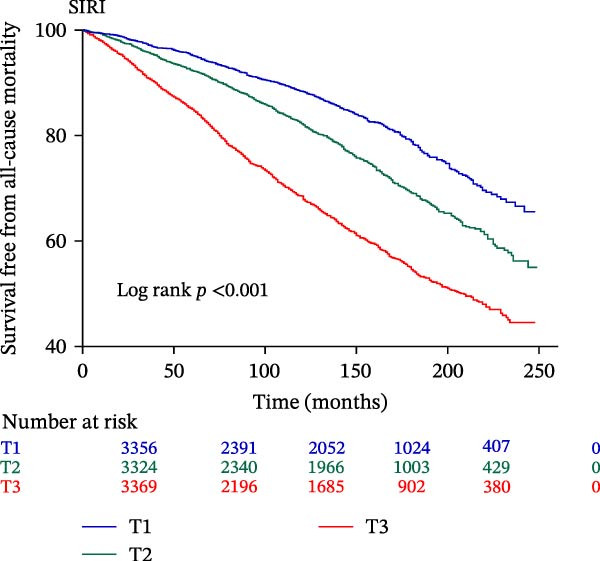
(A)

**Figure:**
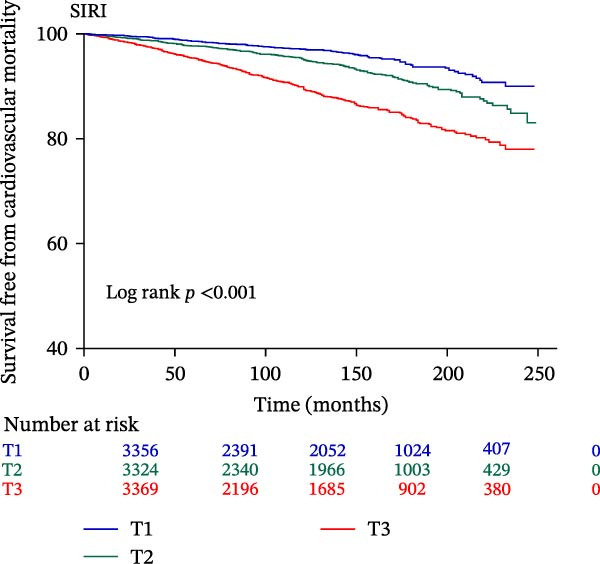
(B)

**Figure:**
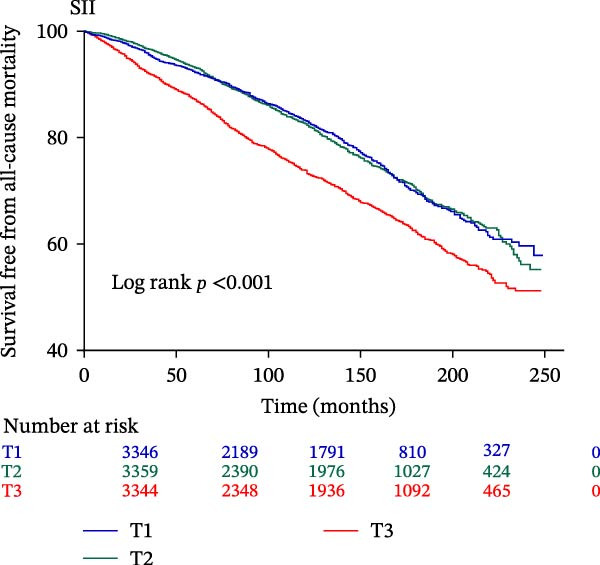
(C)

**Figure:**
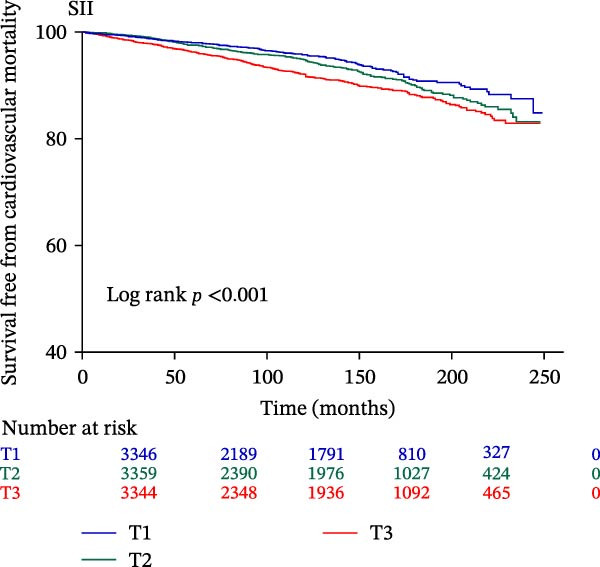
(D)

**Figure:**
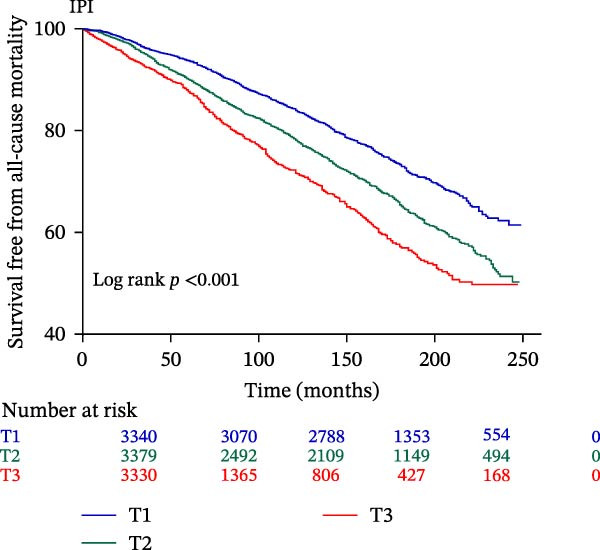
(E)

**Figure:**
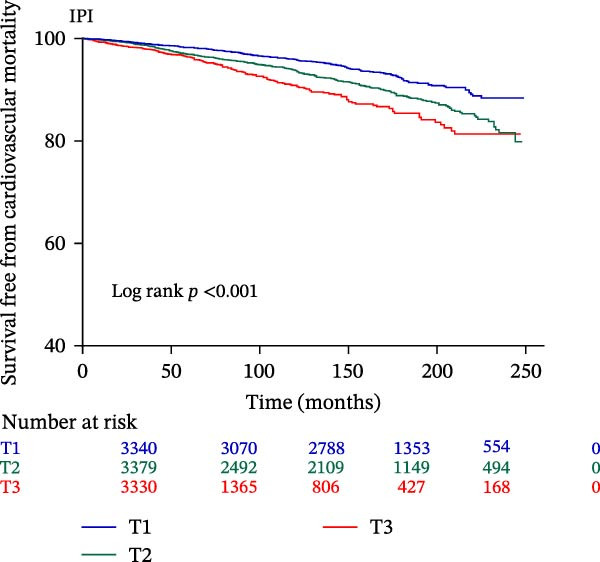
(F)

### 4.3. RCS Regression Analysis of Inflammatory Indices and Mortality Risk

RCS regression was used to model the potentially nonlinear relationships between the inflammatory indices and mortality outcomes (Figure 3). The IPI exhibited a nonlinear correlation with both all‐cause and CVD mortality (all nonlinear *p*  < 0.001) (Figure 3E, F). For the SII, a nonlinear pattern was observed for all‐cause mortality (*p*‐nonlinear < 0.001) (Figure 3C), while its association with CVD mortality followed a linear trend (*p*‐nonlinear = 0.127) (Figure 3D). In contrast, SIRI displayed a linear association with both all‐cause and CVD mortality (nonlinear *p* = 0.118 and *p* = 0.088, respectively) (Figure 3A, B).

Figure 3Spline curves illustrating the associations of SIRI with all‐cause and CVD mortality (A and B), SII with all‐cause and CVD mortality (C and D), and IPI with all‐cause and CVD mortality (E and F) among patients diagnosed with diabetes and prediabetes patients.

**Figure:**
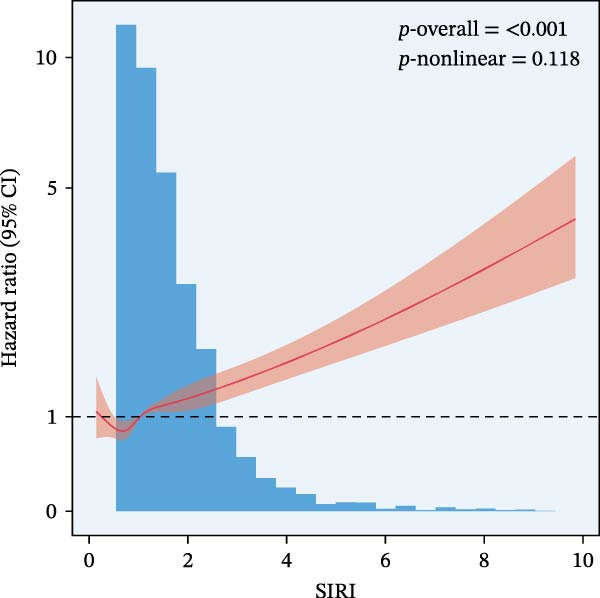
(A)

**Figure:**
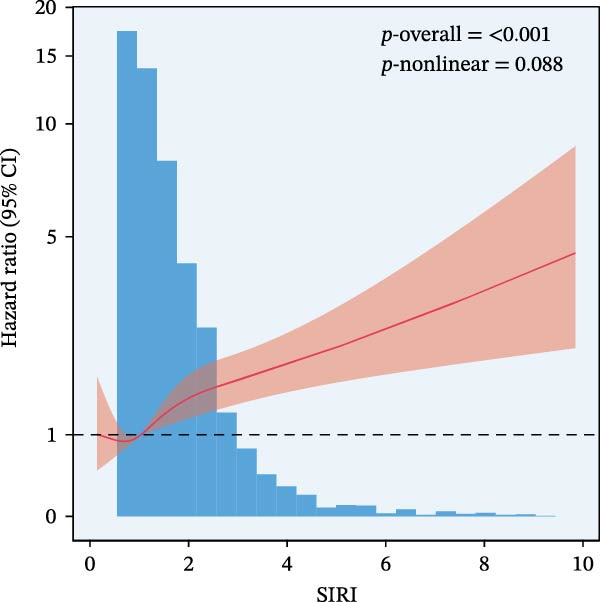
(B)

**Figure:**
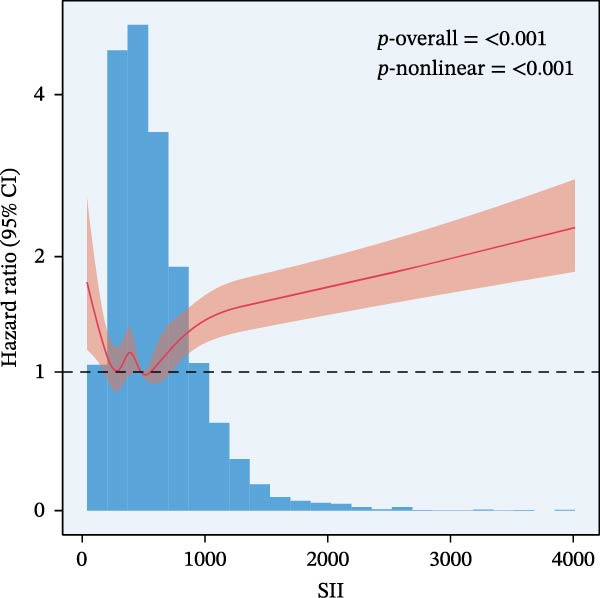
(C)

**Figure:**
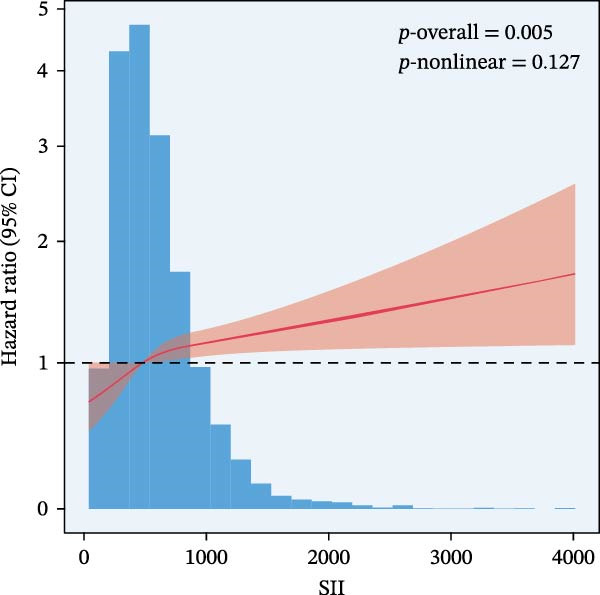
(D)

**Figure:**
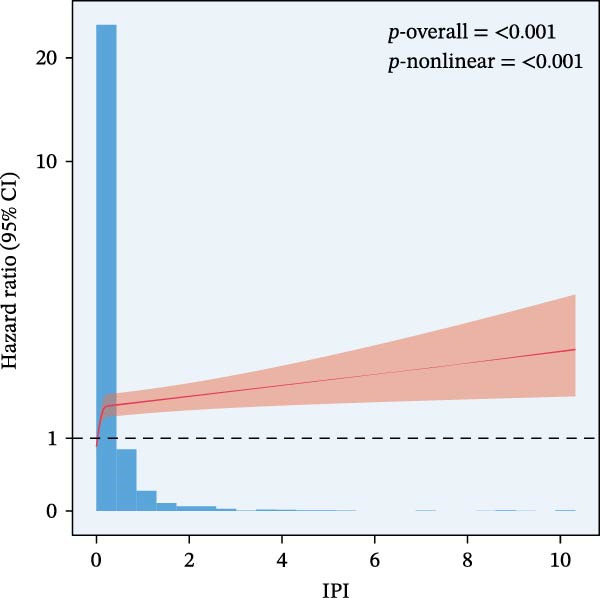
(E)

**Figure:**
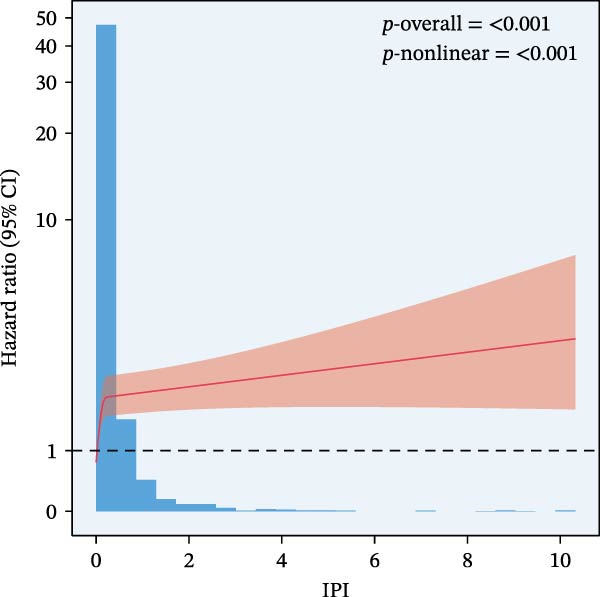
(F)

### 4.4. Subgroup Analyses

We further explored the independent associations of SIRI, SII, and IPI with primary endpoints across specific subgroups (age, sex, history of hypertension, CHD, and CKD) (Table 4). Our findings revealed no significant interactions between SIRI and these subgroups, except for sex, regarding both all‐cause and CVD mortality (interactions *p*  < 0.05). Regarding SII, no significant interactions were found between SII and subgroups, except for age and all‐cause mortality (interaction *p*  < 0.05). As for IPI, we found no significant interactions between IPI and these subgroups, except for sex, which shows significant interactions for both all‐cause and CVD mortality (*p*  < 0.05).

**Table 4:** Subgroup analyses of the correlations between high levels of SIRI, SII, and IPI (T3) with all‐cause and CVD mortality.

Subgroups	All‐cause mortality		CVD mortality	
	HR (95% CI)	*p* for interaction	HR (95% CI)	*p* for interaction
SIRI				
Age	—	0.059	—	0.312
<65	1.748 (1.438–2.123)	—	2.014 (1.377–2.948)	—
≥65	2.159 (1.887–2.470)	—	2.652 (2.028–3.469)	—
Sex	—	0.048	—	0.035
Male	2.775 (2.385–3.229)	—	3.706 (2.722–5.046)	—
Female	2.357 (2.006–2.768)	—	2.510 (1.836–3.433)	—
Hypertension	—	0.690	—	0.935
Yes	2.550 (2.211–2.942)	—	2.991 (2.283–3.917)	—
No	2.451 (2.068–2.905)	—	2.985 (2.082–4.279)	—
CHD	—	0.074	—	0.053
Yes	1.960 (1.390–2.764)	—	1.862 (1.033–3.358)	—
No	3.938 (2.271–2.860)	—	3.124 (2.478–3.940)	—
CKD	—	0.862	—	0.801
Yes	2.431 (1.551–3.809)	—	1.915 (0.901–4.067)	—
No	2.571 (2.297–2.877)	—	3.185 (2.544–3.989)	—
SII				
Age	—	0.006	—	0.192
<65	1.075 (0.892–1.296)	—	1.156 (0.798–1.675)	—
≥65	1.479 (1.307–1.674)	—	1.634 (1.280–2.086)	—
Sex	—	0.936	—	0.138
Male	1.570 (1.376–1.792)	—	1.862 (1.430–2.424)	—
Female	1.382 (1.173–1.628)	—	1.352 (0.984–1.856)	—
Hypertension	—	0.367	—	0.919
Yes	1.490 (1.305–1.703)	—	1.550 (1.207–1.990)	—
No	1.338 (1.139–1.573)	—	1.575 (1.114–2.228)	—
CHD	—	0.477	—	0.143
Yes	1.356 (1.014–1.811)	—	1.126 (0.700–1.811)	—
No	1.477 (1.324–1.649)	—	1.733 (1.383–2.171)	—
CKD	—	0.432	—	0.233
Yes	1.283 (0.888–1.854)	—	1.061 (0.557–2.020)	—
No	1.452 (1.304–1.615)	—	1.634 (1.320–2.024)	—
IPI				
Age	—	0.300	—	0.963
<65	2.014 (1.638–2.475)	—	2.062 (1.357–3.134)	—
≥65	1.775 (1.558–2.021)	—	2.105 (1.650–2.685)	—
Sex	—	0.023	—	<0.001
Male	2.615 (2.260–3.026)	—	3.031 (2.301–3.993)	—
Female	1.243 (1.050–1.472)	—	1.398 (1.006–1.941)	—
Hypertension	—	0.576	—	0.543
Yes	1.614 (1.404–1.856)	—	1.905 (1.473–2.464)	—
No	1.710 (1.428–2.048)	—	1.658 (1.139–2.413)	—
CHD	—	0.135	—	0.391
Yes	2.304 (1.694–3.133)	—	2.405 (1.469–3.938)	—
No	1.758 (1.563–1.977)	—	1.990 (1.576–2.511)	—
CKD	—	0.822	—	0.547
Yes	1.829 (1.168–2.863)	—	2.450 (1.080–5.556)	—
No	1.756 (1.567–1.969)	—	1.940 (1.557–2.418)	—

Abbreviations: CHD, coronary heart disease; CI, confidence interval; CKD, chronic kidney disease; HR, hazard ratio; IPI, inflammatory prognostic index; SII, systemic immune‐inflammation index; SIRI, systemic inflammation response index.

### 4.5. Sensitivity Analysis

Sensitivity analysis was performed to confirm the robustness of our findings. This analysis excluded participants who died within 2 years of follow‐up. In the fully adjusted Cox regression model, the results remained consistent even after excluding participants who deceased within the first 2 years of follow‐up. Specifically, higher SIRI, SII, and IPI values were significantly associated with increased risk of all‐cause mortality (Table 5) and CVD mortality (Table 6), particularly in tertile 3, with significant trends across the tertiles.

**Table 5:** Sensitivity analysis of the associations between SIRI, SII, and IPI with all‐cause mortality.

	Unadjusted		Multivariate adjustment	
	HR (95% CI)	*p*‐Value	HR (95% CI)	*p*‐Value
SIRI				
T1	—	Reference	—	Reference
T2	1.504 (1.326–1.705)	<0.001	1.151 (1.004–1.319)	0.043
T3	2.499 (2.223–2.809)	<0.001	1.429 (1.251–1.632)	<0.001
*p* for trend	—	<0.001	—	<0.001
SII				
T1	—	Reference	—	Reference
T2	1.057 (0.939–1.189)	0.362	0.978 (0.860–1.111)	0.729
T3	1.382 (1.236–1.545)	<0.001	1.156 (1.023–1.307)	0.020
*p* for trend	—	<0.001	—	0.011
IPI				
T1	—	Reference	—	Reference
T2	1.395 (1.260–1.544)	<0.001	1.266 (1.133–1.415)	<0.001
T3	1.667 (1.475–1.883)	<0.001	1.603 (1.394–1.844)	<0.001
*p* for trend	—	<0.001	—	<0.001

*Note:* Multivariate adjusted for age, sex, race, education level, BMI, smoking status, CHD, CKD, HF, stroke, hypertension, TC, TG, HDL, LDL, ALT, AST, and serum creatinine.

Abbreviations: CI, confidence interval; HR, hazard ratio; IPI, inflammatory prognostic index; SII, systemic immune‐inflammation index; SIRI, systemic inflammation response index.

**Table 6:** Sensitivity analysis of the associations between SIRI, SII, and IPI with CVD mortality.

	Unadjusted		Multivariate adjustment	
	HR (95% CI)	*p*‐Value	HR (95% CI)	*p*‐Value
SIRI				
T1	—	Reference	—	Reference
T2	1.596 (1.242–2.050)	<0.001	1.182 (0.899–1.554)	0.230
T3	2.998 (2.382–3.773)	<0.001	1.642 (1.263–2.135)	<0.001
* p* for trend	—	<0.001	—	<0.001
SII				
T1	—	Reference	—	Reference
T2	1.318 (1.048–1.658)	0.018	1.321 (1.028–1.698)	0.029
T3	1.537 (1.230–1.920)	<0.001	1.314 (1.025–1.684)	0.031
*p* for trend	—	<0.001	—	0.046
IPI				
T1	—	Reference	—	Reference
T2	1.560 (1.281–1.899)	<0.001	1.332 (1.071–1.655)	0.010
T3	1.908 (1.511–2.410)	<0.001	1.871 (1.437–2.435)	<0.001
*p* for trend	—	<0.001	—	<0.001

*Note:* Multivariate adjusted for age, sex, race, education level, BMI, smoking status, CHD, CKD, HF, stroke, hypertension, TC, TG, HDL, LDL, ALT, AST, and serum creatinine.

Abbreviations: CI, confidence interval; HR, hazard ratio; IPI, inflammatory prognostic index; SII, systemic immune‐inflammation index; SIRI, systemic inflammation response index.

## 5. Discussion

This large retrospective cohort study highlights the critical role of the IPI in predicting long‐term all‐cause and CVD mortality among individuals with DM and pre‐DM. Our key findings show that higher levels of SIRI, SII, and IPI are strongly linked to increased risks of death. Specifically, participants in the highest third (T3) of each index faced the most significant risk. The Kaplan–Meier survival curves visually support these results, showing a clear difference in survival rates, with the T3 groups experiencing notably poorer outcomes at a median follow‐up of 117 months. Importantly, our RCS analysis uncovered distinct patterns in these associations. While SIRI showed a linear relationship with both types of mortality, SII showed a nonlinear relationship with all‐cause mortality but maintained a linear relationship with CVD mortality. Most notably, IPI revealed a significant nonlinear relationship with both all‐cause and CVD mortality, suggesting that the risk increases more rapidly beyond a certain point. This detailed insight, which is not evident from the simple tertile comparison, highlights the importance of modeling continuous relationships. Additionally, subgroup analysis indicated that sex significantly influenced the association between IPI and mortality, prompting further investigation into sex‐specific inflammatory pathways in diabetes.

DM is acknowledged as the fastest‐growing chronic condition globally, posing a significant risk factor for CVD, CKD, stroke, and several other complications [25, 26]. Pre‐DM was also found to significantly increase the risk of developing DM and may result in complications like nerve damage, vision problems, and kidney disease, typically associated with DM [27]. Both DM and pre‐DM are significant health concerns that warrant attention due to their association with increased mortality risks. A retrospective analysis of 963,648 adults in the US revealed that DM was correlated with a 16% elevation in all‐cause mortality and an 18% rise in CVD mortality [25]. Additionally, a comprehensive study encompassing 2,314,292 individuals demonstrated that the aggregate HR for all‐cause mortality among people with type 2 DM was 2.33 in women and 1.91 in men, compared with the non‐DM population [26]. Similarly, within the general population, individuals with pre‐DM exhibited a 36% heightened risk of all‐cause death, whereas those with pre‐DM showed a 13% increased risk of all‐cause mortality relative to those with normoglycemia [6]. In our study, during a median follow‐up of 9 years, 22.2% of all‐cause mortalities were observed among US adults with DM and pre‐DM, with 612 cases attributed to CVD. Given the elevated mortality risks associated with both DM and pre‐DM, early intervention and preventive approaches are critical to reducing complications and improving long‐term outcomes in these populations.

Chronic inflammation is a key pathogenic feature in both DM and pre‐DM, contributing to insulin resistance and vascular complications [28, 29]. SII, a variable combining platelet and NLR levels, provides a comprehensive assessment of an individual’s inflammatory state and immune status and has been linked to adverse clinical outcomes in conditions such as ischemic stroke, kidney disease, acute coronary syndromes, and cardiogenic shock [11, 30–32]. Similarly, SIRI, calculated from monocyte and NLR levels, has been associated with worse clinical outcomes in patients with conditions such as ischemic stroke, hypertension, and CKD [11, 13, 33]. Systematic studies have confirmed that multiple blood count‐derived inflammatory indices, including SII and SIRI, are significantly associated with all‐cause and CVD mortality in this population, often exhibiting nonlinear relationships and threshold effects [34]. Furthermore, recent clinical evidence supports the role of these indices in diabetes‐related pathology, showing that SII and SIRI levels are elevated in patients with type 2 DM and are positively linked to, and independently serve as risk factors for, insulin resistance [35]. Beyond metabolic disorders, these indices also indicate systemic inflammation that contributes to end‐organ damage, as seen in the association between hyperuricemia and left ventricular hypertrophy [36]. In individuals with DM, multiple clinical studies have demonstrated that an elevated SII is linked with an elevated risk of DM and is closely associated with the risk of CVD and all‐cause mortality. Tang et al. [14] discovered that the SII is closely related to CVD and all‐cause mortality, with variations in these associations observed in participants with varying diabetic statuses. Another study identified a nonlinear association between the SII and risk of death among DM patients [37]. Although previous studies have shown that elevated SIRI levels are associated with a higher risk of diabetic CVD complications [20] and gestational DM [21], the clinical significance of SIRI in the prognosis of DM has not yet been investigated. IPI, derived from CRP, NLR, and albumin, is gaining recognition as a promising prognostic biomarker in cancer patients [22, 38] and has also been shown to predict adverse events among stroke patients [11], as well as for new‐onset atrial fibrillation and mortality following CABG [39]. However, the role of IPI has not been investigated in patients with DM. Our study revealed that, in addition to the established prognostic markers SII and SIRI, the IPI is significantly associated with both all‐cause and CVD mortality in individuals with DM and pre‐DM. The tertile analysis showed a dose–response relationship for IPI, but interestingly, the association for the middle tertile (T2) of SII was not consistently significant across all adjusted models. This may reflect the nonlinear dynamics of systemic inflammation captured by this index. We identified a nonlinear relationship between IPI and mortality outcomes, showing that elevated IPI levels are linked to a higher risk of all‐cause and CVD mortality. Furthermore, we observed a significant interaction between sex and IPI on both all‐cause and CVD mortality, underscoring the unique prognostic value of IPI in this population. The strength of these associations was confirmed by sensitivity analysis excluding early deaths, which produced consistent results. Overall, our study highlights the clinical importance of including comprehensive inflammatory biomarkers in risk assessment models for individuals with DM and pre‐DM. By identifying associations that are independent of traditional risk factors, our findings demonstrate the potential usefulness of these biomarkers in both prognostic evaluation and guiding anti‐inflammatory pharmacological treatments and personalized preventive strategies for patients at increased risk of all‐cause and CVD mortality.

The relationship between elevated risk of mortality and inflammatory biomarkers in individuals with DM and pre‐DM may involve complex underlying pathophysiological mechanisms. Chronic inflammation is a hallmark of DM and pre‐DM, exacerbating insulin resistance and vascular dysfunction [40]. Elevated SII, SIRI, and IPI levels reflect the complex interplay between various inflammatory biomarkers, including neutrophils, monocytes, lymphocytes, platelets, albumin, and CRP, which are implicated in the progression of CVD and all‐cause death. The nonlinear relationships observed between SII and all‐cause mortality, and between IPI and all‐cause and CVD mortality, suggest multifaceted interactions between inflammation and disease progression in DM and pre‐DM. Further analysis across different ethnicities is warranted to confirm these findings, which could provide valuable insights into potential ethnic‐specific differences in the associations between inflammatory biomarkers and prognosis in DM and pre‐DM populations.

From a clinical standpoint, the IPI, a composite score derived from routine blood tests (CRP, NLR, and albumin), is a practical, cost‐effective tool for risk stratification. Our findings, especially the nonlinear threshold effect, suggest that it could be incorporated into standard patient management for those with DM and pre‐DM to identify those at the highest mortality risk. Individuals with an IPI above the identified inflection point may benefit from closer monitoring, lifestyle modifications, or assessment for additional anti‐inflammatory treatments. The significant interaction with sex also indicates the possible need for sex‐specific risk thresholds. Future studies should validate these thresholds in independent cohorts and examine whether reducing IPI through medication or lifestyle changes improves clinical outcomes, thereby shifting this biomarker from a prognostic tool to a guide for therapy.

The current study has several strengths, including its large sample size with both DM and pre‐DM populations. In addition to established inflammatory biomarkers such as SIRI and SII, which are known to have prognostic value in DM, this study incorporates the IPI, calculated as CRP × NLR/albumin. Elevated levels of IPI are associated with higher all‐cause and CVD mortality in patients with DM and pre‐DM, providing an additional layer of prognostic value by integrating critical inflammatory markers. The comprehensive data from NHANES allowed for the adjustment of a wide range of potential confounding factors, including age, sex, race, education level, BMI, smoking status, and comorbidities such as CHD, CKD, HF, stroke, and hypertension, along with lipid profiles, liver function markers, and serum creatinine, further supporting the robustness of the findings. Several limitations must be acknowledged. First, our study design precluded establishing a causal relationship, and the ethnic diversity represented solely by the US population restricts the generalizability of our findings to other global populations. Second, the severity and duration of diabetes could not be adequately assessed due to insufficient information in the dataset; specifically, data on diabetes duration were not available. Third, despite controlling for numerous confounders, unaccounted factors that could affect our conclusions may still exist. Finally, several methodological considerations for assessing inflammation should be acknowledged. The inflammatory indices are based on a single measurement, which may capture both chronic low‐grade and transient acute inflammatory states. Furthermore, baseline acute or chronic localized inflammatory conditions were not systematically assessed. Consequently, we cannot definitively attribute the observed mortality associations solely to persistent, low‐grade systemic inflammation. Additionally, the composite indices integrate biomarkers measured from different sample matrices (whole blood for cellular components and serum for proteins), which, while standard, is a nuance to consider when interpreting these scores as unified inflammatory measures.

## 6. Conclusion

In this large cohort study with a median follow‐up of 9 years, we observed that elevated IPI levels were significantly associated with increased all‐cause and CVD mortality among individuals with DM and pre‐DM. These findings highlight the potential of IPI as a novel, comprehensive biomarker for risk stratification. However, these results should be interpreted with caution and need validation in independent, diverse populations. Future research is necessary to confirm its prognostic utility and to define its role in guiding personalized preventive strategies and anti‐inflammatory treatment in clinical practice.

**Nomenclature**
DM: Diabetes mellitus
pre‐DM: Prediabetes
IPI: Inflammatory prognosis index
HbA1c: Hemoglobin A1c
CVDs: Cardiovascular diseases
SIRI: Systemic inflammatory response index
SII: Systemic immune‐inflammation index
NHANES: National Health and Nutrition Examination Survey
CRP: C‐reactive protein
NLR: Neutrophil‐to‐lymphocyte ratio
FBG: Fasting blood glucose
CKD: Chronic kidney disease
HF: Heart failure
CHD: Coronary heart disease.

## Author Contributions

**Lu Liu:** writing – original draft, conceptualization, investigation. **Fuad A. Abdu:** data curation, formal analysis, supervision, writing – review and editing. **Jiasuer Alifu:** writing – review and editing, formal analysis. **Abdul-Quddus Mohammed:** writing – original draft, data curation. **Guoqing Yin:** writing – review and editing, conceptualization. **Wenliang Che:** writing – review and editing, supervision, conceptualization.

## Funding

This work was supported in part by the Chinese National Natural Science Foundation (Grant 82170521), the Shanghai Natural Science Foundation of China (Grant 21ZR1449500), the Foundation of Shanghai Municipal Health Commission (Grant 202140263), the Tibet Natural Science Foundation of China (Grants XZ2022ZR‐ZY27(Z) and XZ202301ZR0032G), the Foundation of Chongming (Grants CKY2021‐21 and CKY2020‐29), the Clinical Research Plan of Shanghai Tenth People’s Hospital (Grant YNCR2A001), the Shanghai Hospital Development Center Foundation (Grant SHDC12024120), the Clinical Research Plan of SHDC (Grant SHDC2020CR4065), and the Foundation of the Science and Technology Commission of Shanghai Municipality (Grant 20dz1207200).

## Ethics Statement

All surveys were thoroughly reviewed and approved by the National Center for Health Statistics Ethics Review Committee, and all participants provided written informed consent prior to participation.

## Consent

The authors have nothing to report.

## Conflicts of Interest

The authors declare no conflicts of interest.

## Data Availability

The data that support the findings of this study are available from the corresponding author upon reasonable request.
